# Current Enlightenment About Etiology and Pharmacological Treatment of Autism Spectrum Disorder

**DOI:** 10.3389/fnins.2018.00304

**Published:** 2018-05-16

**Authors:** Nermin Eissa, Mohammed Al-Houqani, Adel Sadeq, Shreesh K. Ojha, Astrid Sasse, Bassem Sadek

**Affiliations:** ^1^Department of Pharmacology and Therapeutics, College of Medicine and Health Sciences, United Arab Emirates University, Al Ain, United Arab Emirates; ^2^Department of Internal Medicine, College of Medicine and Health Sciences, United Arab Emirates University, Al Ain, United Arab Emirates; ^3^Department of Clinical Pharmacy, College of Pharmacy, Al Ain University of Science and Technology, Al Ain, United Arab Emirates; ^4^School of Pharmacy and Pharmaceutical Sciences, Trinity College Dublin, University of Dublin, Dublin, Ireland

**Keywords:** autistic spectrum disorder, genetic factors, environmental factors, neurotransmitter dysfunctions, neurodegeneration, neuroprotection, cognitive deficits, pharmacological intervention

## Abstract

Autistic Spectrum Disorder (ASD) is a complex neurodevelopmental brain disorder characterized by two core behavioral symptoms, namely impairments in social communication and restricted/repetitive behavior. The molecular mechanisms underlying ASD are not well understood. Recent genetic as well as non-genetic animal models contributed significantly in understanding the pathophysiology of ASD, as they establish autism-like behavior in mice and rats. Among the genetic causes, several chromosomal mutations including duplications or deletions could be possible causative factors of ASD. In addition, the biochemical basis suggests that several brain neurotransmitters, e.g., dopamine (DA), serotonin (5-HT), gamma-amino butyric acid (GABA), acetylcholine (ACh), glutamate (Glu) and histamine (HA) participate in the onset and progression of ASD. Despite of convincible understanding, risperidone and aripiprazole are the only two drugs available clinically for improving behavioral symptoms of ASD following approval by Food and Drug Administration (FDA). Till date, up to our knowledge there is no other drug approved for clinical usage specifically for ASD symptoms. However, many novel drug candidates and classes of compounds are underway for ASD at different phases of preclinical and clinical drug development. In this review, the diversity of numerous aetiological factors and the alterations in variety of neurotransmitter generation, release and function linked to ASD are discussed with focus on drugs currently used to manage neuropsychiatric symptoms related to ASD. The review also highlights the clinical development of drugs with emphasis on their pharmacological targets aiming at improving core symptoms in ASD.

## Introduction

Autistic spectrum disorder (ASD) is a biologically based neurodevelopmental disorder affecting two major core behavioral symptoms, namely impairments in social skills and restricted /repetitive behavioral pattern or interest of ASD patients (Baronio et al., 2015). These core symptoms can be observed before the age of three years and are lasting for the whole lifetime (Andres, 2002). ASD has become high priority for scientists and health care providers, and has also attracted the public attention because of reported increase in its prevalence (Sheldrick and Carter, 2018; Xu et al., 2018). The worldwide estimated prevalence of individuals with ASD diagnosis is strikingly high with prevalence varying across numerous studies, but it is estimated that one in 160 children has an ASD worldwide, and it is expected to increase globally (Arvidsson et al., 2018). Despite its increasing prevalence, the pathophysiology of ASD is still understood incompletely, and this can be attributed to challenges in identifying suitable animal models and the complexity of the neurobiology in brain function (Nestler and Hyman, 2010). Several evidences suggest that strong genetic and environmental factors rise the occurrence of ASD in childhood (Baronio et al., 2015). To date, there are no efficient therapeutic interventions that target the core symptoms of ASD, namely social communication impairments and restricted/repetitive behavior (Sheldrick and Carter, 2018; Xu et al., 2018). However, pharmacological interventions may be used to provide symptomatic control of associated comorbidities but not to treat core deficits (Wong and Smith, 2006; Hanson et al., 2007). Notably, it is nowadays believed that ASD and schizophrenia (SCH) are conceptualized as two separate disorders, despite the fact that both ASD and SCH were found to share multiple aetiologies, phenotypic feature similarities, and risk factors, and were reported to co-occur at elevated rates (Chisholm et al., 2015).

## Etiology of ASD

ASD is broadly considered to be a multi-factorial disorder that results from genetic as well as non-genetic risk factors. There is cumulative evidence for the involvement of genetic factors in the etiology of ASD, since siblings born in families with ASD are at 35–40% greater risk to develop ASD and with an increase in the current rate of approximately 1% from a rate of 0.05% in 1970s (Stubbs et al., 2016). Moreover, genetic studies revealed that alteration in the developmental pathways of neuronal and axonal structures that are strongly involved in synaptogenesis emerge from single gene mutations (Geschwind, 2011; Voineagu et al., 2011; Chang et al., 2015). It is likely that interactions between multiple genes, and variability in expression as a result of epigenetic factors and exposure to environmental factors are responsible for ASD (Muhle et al., 2004). In a previous clinical study involving a twin, it was appraised that the risk of developing ASD was 35–40% due to genetic variability, and the remaining 60% was contributed to by prenatal, perinatal, and postnatal environmental factors (Hallmayer et al., 2011). Accordingly, environmental factors implicated with ASD included prenatal and perinatal complications (Glasson et al., 2004; Maramara et al., 2014), birth and neonatal complications (Gardener et al., 2011; Guinchat et al., 2012), viral infection, autoimmune diseases, and exposure to teratogens and maternal anticonvulsants such as valproic acid (VPA) (Kern and Jones, 2006; Kolevzon et al., 2007). Therefore, an increased understanding of the interface between genetic and environmental factors in the pathogenesis of ASD may lead to an optimized therapeutic strategy.

## Correlation of neurotransmitters dysfunction to ASD

**Figure 1:**
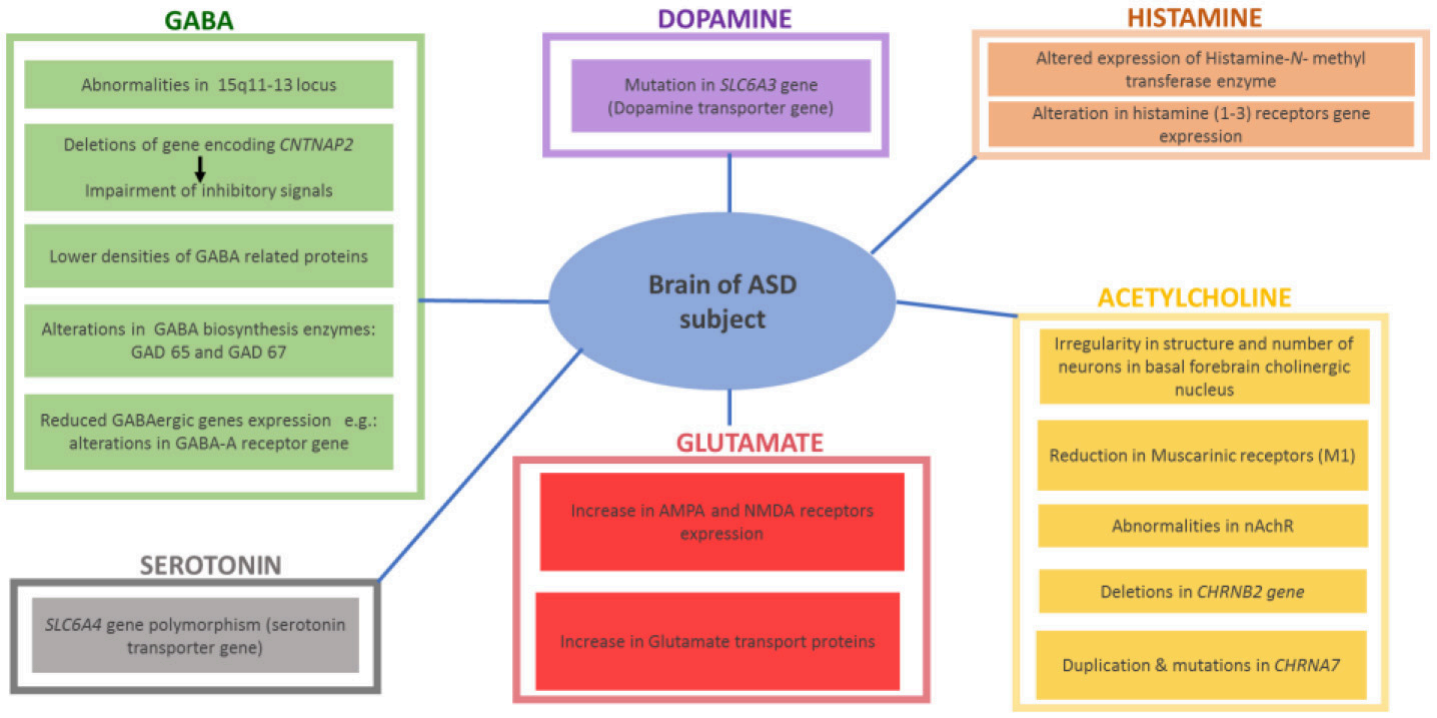
Reported findings in different brains of ASD patients with altered central neurotransmitters.

Research has also focused on the study of neurotransmitters, in search of sensitive and specific markers of ASD as well as potential therapeutic interventions. The role of several central neurotransmitters (e.g., 5-HT, ACh, DA, GABA and Glu) in initial brain development may substantiate to be a significant area in studying the etiology of ASD. Certain disruption of brain neurotransmissions early during the development phase of the CNS may demonstrate early pharmacological intervention that helps to cure and maybe even preclude some of the severe behavioral symptoms of ASD. Ideally, the work in genetics may be able to explain these neurochemical defects at birth, providing possible appropriate medical treatment for infants who are at increased risk for ASD. This would completely exhibit new therapeutic tactic to the clinical control of ASD. Growing evidences suggest that a variety of several neurotransmitter systems such as ACh, 5-HT, DA, GABA, Glu, and HA are implicated in the onset and progression of ASD -along with genetic and environmental factors discussed below- (Shah and Wing, 2006; Bacchelli et al., 2015; Ellenbroek and Ghiabi, 2015; Wang et al., 2015; Chen et al., 2017; Hellings et al., 2017; Hellmer and Nystrom, 2017; Naaijen et al., 2017; Nakai et al., 2017; Paval, 2017; Paval et al., 2017; Figure 1).

### Serotonin

Among all neurotransmitters investigated so far in ASD, 5-HT has motivated the most research efforts and investigations. 5-HT signaling facilitates several neural processes including neurogenesis, cell migration and survival, synaptogenesis, and synaptic plasticity. Interestingly, high 5-HT levels in the blood have been described for up to 45% of tested ASD subjects(Chen et al., 2015, 2017; Ellenbroek et al., 2016). Moreover, preclinical investigations using ASD-like animal models reported that hyperserotonemia significantly reduced the motivation for social interest through inhibition of separation distress, potentially accounting for the social impairments found in ASD individuals (Ellenbroek et al., 2016; Nakai et al., 2017). Furthermore, 5-HT was found to accumulate mainly in platelets utilizing the specific 5-HT transporter. In line with these findings, genetic studies of linkage stated that the 17q11.2 region containing the 5-HT transporter gene *SLC6A4* polymorphisms appear to be associated with ASD, as Gly56 conversion to Ala56 in the transporter protein resulted in autistic phenotypic features combined with an amplified p38-mitogen-activated protein kinases (MAPK)-sensitive basal phosphorylation process. In addition and in a previous study, higher clearance rates of hippocampal 5-HT were observed and hence hyperserotonemia, which led to a significant hypersensitivity of brain 5-HT(1A) as well as 5-HT(2A) receptors, social impairment and repetitive behavior (Veenstra-VanderWeele et al., 2012; Figure 1).

### Dopamine

Dopamine (DA) plays a fundamental role in brain functioning, and the pathophysiological role of dopaminergic system (DS) deficits in ASD is well recognized, with the wide clinical use of antipsychotics that mainly target the D2 receptors (D2Rs)(Seeman, 2010; Baronio et al., 2014) Interestingly, and in a very recent preclinical study, it has been shown that mice with increased dopaminergic neurotransmission in the dorsal striatum via the suppression of dopamine transporter expression in substantia nigra neurons or the optogenetic stimulation of the nigro-striatal circuitry exhibited significant deficits in sociability and repetitive behaviors relevant to ASD pathology in several rodent models, while these behavioral changes were blocked by using D1R antagonists (Lee et al., 2017). Therefore, D1R agonists produced typical autistic-like behaviors in normal mice or the genetic knockout (KO) of D2Rs (Lee et al., 2017). Furthermore, the siRNA-mediated inhibition of D2Rs in the dorsal striatum was shown to replicate ASD-like phenotypes in D2R KO mice (Lee et al., 2017). With regard to the DS, genetic studies have demonstrated that mutations of DS-associated genes such as the DA transporter (DAT) (Hamilton et al., 2013), DA receptors (Hettinger et al., 2008; Qian et al., 2013), and enzymes of DA biosynthesis (Nguyen et al., 2014) are implicated in ASD. These studies expanded the evidences of genetically linking between DA transporter and ASD (Hamilton et al., 2013; Bowton et al., 2014). ASD is strongly associated with a mutation in the DA transporter gene *SLC6A3*, which codes for a protein that contributes to regulation of DA levels in the brain. In fact, DAT is the crucial regulator of DA homeostasis and alteration of this homeostasis as a consequence of DAT dysfunction is associated with ASD and other neuropsychiatric conditions (Hamilton et al., 2013; Hellings et al., 2017; Paval, 2017; Paval et al., 2017). Moreover, it has been found that the dopaminergic fibers arising from the ventral tegmental area (VTA) project to the prefrontal cortex (PFC) and to regions of the limbic system, such as the nucleus accumbens (NAcc), forming the mesocorticolimbic (MCL) circuit. This circuit was found to be involved in high order brain functions, such as emotional social behavior, reward, motivation and cognition (Paval et al., 2017) (Hellings et al., 2017; Figure 1). Notably, the DS has been related to behavioral skills belonging to executive functioning, such as analyzing, planning and prioritizing (Seeman, 2010; Baronio et al., 2014). In line with that, children diagnosed with ASD were found to show deficits in tasks linked to executive functioning, including response/selection, planning/working memory and flexibility (Hellings et al., 2017; Paval et al., 2017). Moreover, the DS has strongly been linked to social behavior, attentional skills and perception, and motor activity, while development abnormalities in these areas have all been linked to ASD as well (Hellings et al., 2017; Paval et al., 2017).

### GABA and glutamate

Gabaminergic as well as glutaminergic systems are also proposed as potential mechanisms for ASD. Consequently, mutations in the respective synaptic proteins would lead to defective neurotransmissions at excitatory and inhibitory synapses, leading to disruption of excitatory-inhibitory balance of neurotransmissions in postsynaptic neurons, a key mechanism which has strongly been associated with ASD (Jamain et al., 2002; Naaijen et al., 2017). In addition to numerous reports stating the duplication of 15q11-13 locus in ASD populations, a majority of the cases do not show mutations at this locus. However, they do exhibit abnormalities in the expression of protein encoded by 15q11-13 gene, as reported. These results demonstrated the involvement of 15q11-13 locus in ASD, either directly by mutation or indirectly by epigenetic factors (Hogart et al., 2007; Nakai et al., 2017). This site employs numerous genes coding for particular subunits of GABA receptors, namely GABRB3, GABRA5 and GABRG3. There are numerous signaling and scaffolding proteins which are implicated in normal development and GABA synapse function. Therefore, mutations in the genes encoding these proteins consequently result in GABAergic dysfunction, hence inhibitory signaling deficits. For example, deletions in gene encoding a protein contactin-associated protein 2 (CNTNAP2) has been linked to ASD (Stephan, 2008; Gregor et al., 2011; Nord et al., 2011). Moreover, deficiency in CNTNAP2 results in reduction in GAD1, parvalbumin and inhibitory interneurons along with impairments in inhibitory signaling (Peñagarikano et al., 2011; Soghomonian et al., 2017). This evidence suggests an association and the relation between mutations affecting the function of GABA and ASD. Furthermore, reduced expression of GABAergic genes and lower density of GABA related proteins have been found in individual brains of ASD patients. Consequently, alterations in GABA-A receptor genes and other genes expressed on GABA interneurons, as well as GABA biosynthesis enzymes (GAD 65 and GAD 67) in the cerebellum and parietal cortex, are strongly implicated in ASD (Fatemi et al., 2002; Coghlan et al., 2012). On the contrary, an increased level in the expression of excitatory Glu receptor AMPA, and of Glu transporter proteins were observed in individuals diagnosed with ASD, with highest expression abnormalities found in the cerebellum (Purcell et al., 2001; Figure 1). Based on the aforementioned, altered expression of genes related to GABA and/or Glu might be linked to several ASD phenotypic features including cognitive deficits and/or hyperactivity (Jamain et al., 2002; Naaijen et al., 2017). Moreover, abnormalities in both neurotransmitters including an increase in the excitatory Glu and/or decrease in the inhibitory GABA were found to lead to epileptic seizure, a clinical feature which is commonly observed in ASD patients (Ballaban-Gil and Tuchman, 2000; Levisohn, 2007; Gillberg et al., 2017).

### Acetylcholine

The brain cholinergic neurotransmission system with ACh plays an essential role in regulating ASD-related behavioral symptoms including attention (Arnold et al., 2002), cognitive flexibility (Ragozzino et al., 1998), social interaction (Avale et al., 2011) and stereotypical behaviors (McConville et al., 1992; Bacchelli et al., 2015; Wang et al., 2015; Hellmer and Nystrom, 2017). Mounting evidences suggest the involvement of cholinergic system dysfunction in the phenotypic outcomes of ASD-related behavioral features, in both humans and animal models - (Karvat and Kimchi, 2014). In ASD patients, there are remarkable abnormalities in the cholinergic system. Anatomically there is irregularity in the number and structure of neurons in a basal forebrain cholinergic nucleus of patients diagnosed with ASD (Kemper and Bauman, 1998). Also, a decrease in the level of choline, the precursor of the neurotransmitter ACh and the agonist for nicotinic-cholinergic receptor (nAChR), was determined in individuals diagnosed with ASD (Friedman et al., 2006), with the severity of ASD related to the decreased level of cytosolic ACh (Sokol et al., 2002). Moreover, and by using immunohistochemical analyses, abnormalities in nAChR were observed in several brain regions (e.g., neocortex, cerebellum, thalamus and striatum) of ASD patients, specifically with the major abnormalities being reduction in nAChR subunits and muscarinic receptors (M1 type) (Mukaetova-Ladinska, 2017). Moreover, the brain ACh like several other brain neurotransmitters, e.g., DA, 5-HT and GABA, playing a key role in ASD are regulated by different central mechanisms including histamine H3 hetero-receptors (H3Rs), highlighting the attention to brain histaminergic system involvement in the ASD (Sadek and Stark, 2015; Sadek et al., 2016; Alachkar et al., 2017; Eissa et al., 2018). Genetically, studies reported duplications and mutations in *CHRNA7*, the gene encoding the α7nAChR subunit, in ASD patients (Mikhail et al., 2011; Leblond et al., 2012), whereas deletion of the *CHRNB2* gene, encoding the β2-subunit of nAChR was observed in other cases (Granon et al., 2003). Furthermore, ASD related behavior may be linked to M1 type mAChR inhibition (McCool et al., 2008) and cholinergic cell damage (Walker et al., 2007; Figure 1). Consequently, social deficits and repetitive behaviors are the main phenotypic ASD features connected to disruption in cholinergic neurotransmission system (Wang et al., 2015). Also, reduced attention (Arnold et al., 2002), decreased cognitive flexibility (Ragozzino et al., 1998), reduced social communications (Avale et al., 2011) and conventional behaviors have been strongly linked to cholinergic neurotransmission dysfunction (McConville et al., 1992; Bacchelli et al., 2015; Wang et al., 2015; Hellmer and Nystrom, 2017).

### Histamine

**Figure 2:**
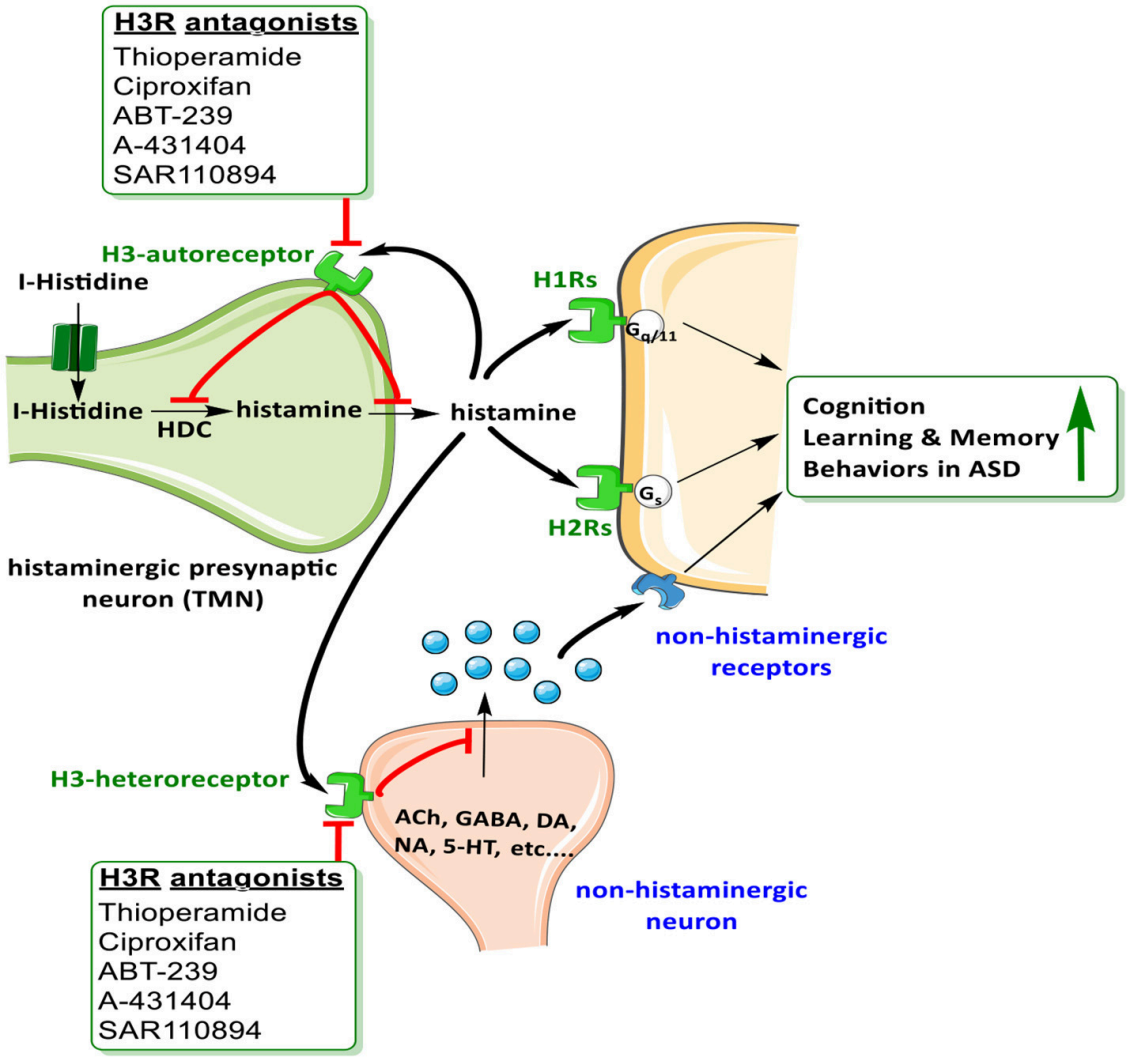
Schematic illustration of possible mechanisms of H3R antagonists on H3-auto receptors and hetero-receptors.

The brain histaminergic system was found to display a critical role in cognition, sleep and other neuropsychiatric disorders including schizophrenia (SCH) and Tourette syndrome that share comorbidity with ASD (Wright et al., 2017). Moreover, alteration in gene expression was found for histamine-*N*-methyltransferase enzyme (HNMT, enzyme responsible for metabolism of central histamine (HA) and for histamine receptor (HR) subtypes H1-, H2-, and H3R (Wright et al., 2017). Notably, there has in the last two decades been a rising interest in studying the role of brain HA on behaviors in both physiological conditions and psychiatric diseases, e.g., SCH (Sadek and Stark, 2015; Sadek et al., 2016). Interestingly and as revealed in several clinical reports, patients with SCH shared a variety of common symptoms and genetic factors with ASD (Konstantareas and Hewitt, 2001; Carroll and Owen, 2009). Moreover, a significant role for the brain HA has been projected, and a range of several H3R ligands has been developed so far for dual-targeting of both dopaminergic as well as histaminergic neurotransmissions (Bishara, 2010; Baronio et al., 2014). Furthermore and as discussed above, deficits in regulations of various other neurotransmitters including DA, 5-HT, GABA, and Glu are postulated (Witkin and Nelson, 2004). Therefore, the role of central HA that influences behavior in CNS disorder sheds light on the histaminergic system as pharmacological target for therapeutic purposes (Haas et al., 2008; Tiligada et al., 2011; Shan et al., 2012; Naddafi and Mirshafiey, 2013). Interestingly, brain H3Rs act as auto-receptors or hetero-receptors that regulates the biosynthesis and release of HA and several other neurotransmitters, which consequently play a role in cognitive and homeostatic processes, as shown in Figures 1, 2. Therefore, there is an indirect indication that histaminergic neurotransmission may have a significant role in SCH and that potent and selective H3R antagonists could lead to therapeutic improvements of cognitive symptoms associated with SCH and ASD (Witkin and Nelson, 2004; Esbenshade et al., 2008; von Coburg et al., 2009; Brown et al., 2013; Sadek et al., 2016). To date there are scarce literature studies concerning the association of H3R antagonists and treatment of ASD behavioral deficits. Accordingly and in a previous preclinical study, the imidazole-based H3R antagonists, namely thioperamide and ciproxifan, improved the decreased prepulse inhibition in an animal model of SCH (Brown et al., 2013; Figure 2, Table 4). Thioperamide and ciproxifan are selective and potent imidazole-based H3R antagonists that are widely used in preclinical animal experiments (Ligneau et al., 1998; Stark et al., 2000). However, the preclinical use of numerous non-imidazole-based H3R antagonists, e.g., ABT-239 and A-431404, ameliorated ketamine- and/or MK-801-induced cognitive impairments in experimental rats, demonstrating enhanced results when compared with reference antipsychotics like risperidone or olanzapine (Brown et al., 2013; Figure 2, Table 4). Interestingly, H3R antagonists possess antioxidant activity which could increase their potential clinical use, since oxidative stress is, also, considered as possible predictors of intensified symptoms of SCH and ASD (Mahmood et al., 2012). Moreover, a study considering the effectiveness of the non-imidazole H3R antagonist ABT-288 in the treatment of cognitive deficits linked with SCH revealed that schizophrenic features remained constant during the whole time period of the study, with acceptable safety, tolerability and pharmacokinetics profile of ABT-288 at a 15-fold higher dose and 12-fold higher exposures in subjects with SCH than previously observed in healthy volunteers (Coruzzi et al., 2012) (Hsieh et al., 2010). Moreover, the use of a H3R antagonist ameliorated behavioral impairments in an animal model of SCH, including spatial working memory deficit, an abnormality which also characterizes patients with ASD (Steele et al., 2007). Also, a recent preclinical study reported that an acute systemic administration of ciproxifan palliated some sociability impairments and stereotypic behavior in an animal model of VPA-induced ASD in mice (Baronio et al., 2015). However, further research efforts are still necessary to magnify these observed initial data and to achieve enhanced understanding of pathophysiology and therapeutic management of ASD. Based on the aforementioned observations, numerous brain neurotransmitters, e.g., 5-HT, DA, GABA, Glu, ACh, and HA appear to be implicated in the pathophysiology of ASD, since disruption of genes encoding different proteins (receptors and/or catalyzing biosynthesis enzymes for respective neurotransmitter) that affect the respective neurotransmission functionality leads to a variety of phenotypic features of ASD. Interestingly, the above discussed dysfunctions of neurotransmitters, namely 5-HT, DA, GABA, Glu, ACh, and HA were, also, found to be influentially implicated in the clinical outcome features of SCH, a disorders that is comorbid and shares multiple etiologies and risk factors with ASD (Chisholm et al., 2015; Devor et al., 2017).

## Current pharmacological intervention

**Figure 3:**
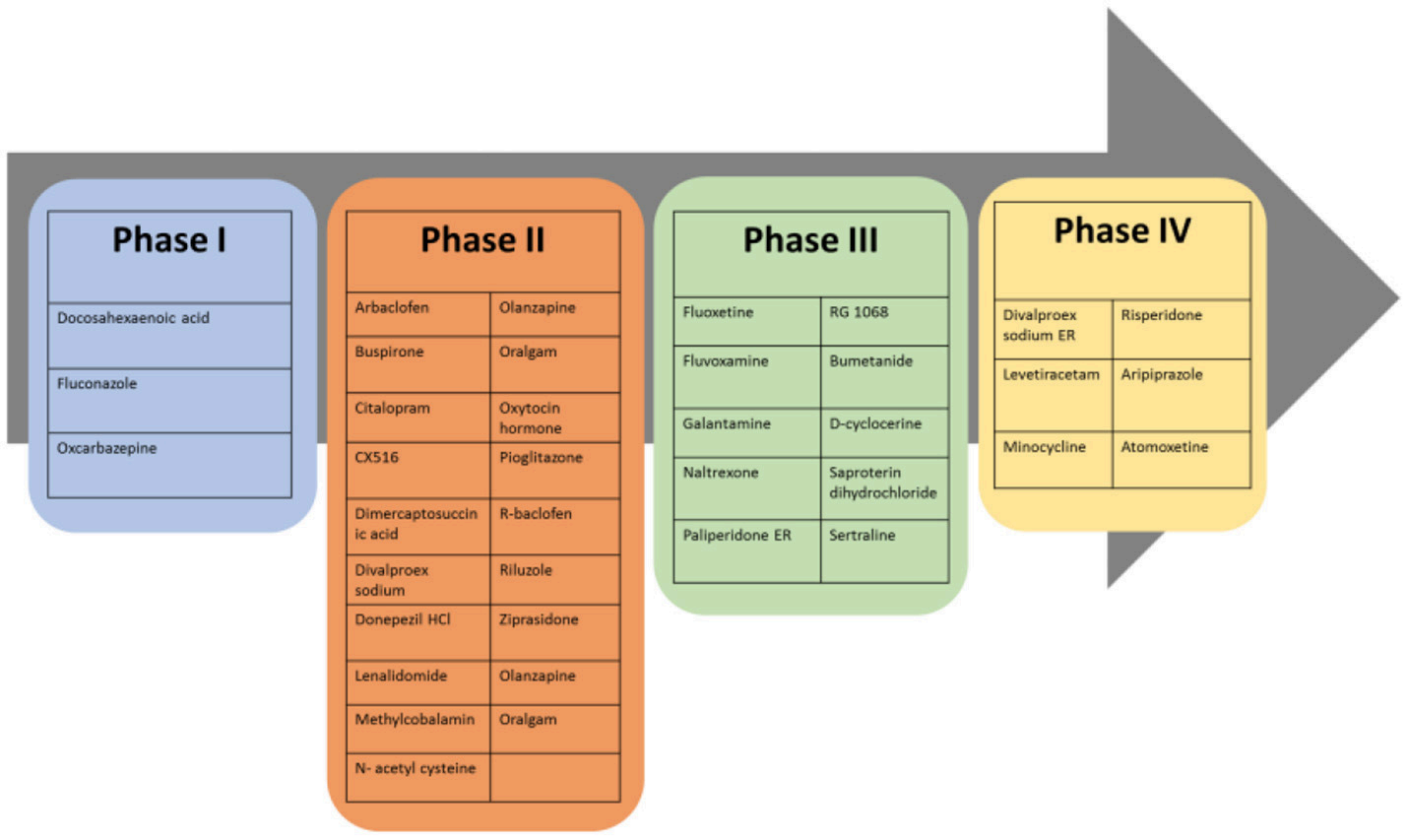
Most promising drugs at different clinical trial phases for future therapeutic management of ASD behavioral and neurological symptoms.

Based on the aforementioned abnormalities in genes as well as various neurotransmitter systems, studying the effects of a given drug on core symptoms in ASD is very challenging. Despite advances in early diagnosis and intervention, efficacious reversal of core autistic symptoms is still not accomplished to date. At present time there is no definite pharmacological treatment for ASD but treatments for ASD are based on behavioral therapies and the use of highly controlled learning environments. The recent approaches to treatment of ASD set behavioral therapy and atypical language development, as keystone for ASD therapy along with other treatments that tends to ameliorate associated symptoms and not the core deficits (Sathe et al., 2017; Weitlauf et al., 2017). The heterogeneity of clinical and behavioral features in children diagnosed with ASD contributes to the difficulty in understanding the pathophysiology of this disorder, and consequently, no specific treatment can be effective for all ASD children. Therefore, subgrouping of children based on responses to intervention is essential (James et al., 2009). As mentioned above, targeting ASD core symptoms for complete and effective treatment has been challenging and not yet achieved, however several pharmacological medications maybe effective in various associated symptoms that often cause significant impairments in ASD (Volkmar et al., 1999). These associated symptoms of ASD include inattention, hyperactivity, anxiety, sleep disturbances, irritability, repetitive behavior, aggression and self-injury. Antipsychotics are often used for therapeutic management of ASD symptoms in children (Findling et al., 2004). Currently, atypical antipsychotics risperidone and aripiprazole are the only two drugs which have so far been approved by FDA for improving behavioral symptoms associated with ASD (Matson et al., 2011), however, there are several other pharmacological interventions that show effective clinical management of ASD symptoms. The most promising drugs reported for managing behavioral and neurological symptoms of ASD are acting on different brain targets and are summarized in Table 1. Accordingly, therapeutic benefits have been observed and described with several classes of drugs including selective serotonin reuptake inhibitors (sertraline, citalopram, fluoxetine) for anxiety and repetitive behaviors, psychostimulant (methylphenidate) for hyperactivity, opioid antagonist (naltrexone) for hyperactivity, and atypical antipsychotics (risperidone, olanzapine, clozapine) for temper tantrums, aggression, or self-injurious behavior (Aman, 2004; Moore et al., 2004; Kumar et al., 2012). Notably, numerous candidates of the classes discussed below have progressed to several phases of clinical trials as shown in Figure 3.

**Table 1:** Selected medications of different classes that are currently used to manage different ASD symptoms.

**Class**	**Medication**	**Structure**	**Pharmacological effects**	**Side effects**	**Reference**
**Atypical Antipsychotics**	Clozapine	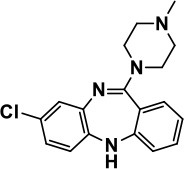	Improves hyperactivity and aggression in ASD patients	Requires patient's hematological safety monitoring and it lowers seizure threshold	Zuddas et al., 1996; Chen et al., 2001; Gobbi and Pulvirenti, 2001; Sahoo et al., 2017
	Risperidone	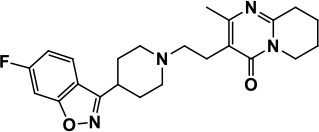	Reduces irritability, repetitive behavior, aggression, anxiety, and depression & nervousness. It shows neuroprotective activity, modulates astroglia function, and increases the brain antioxidant activity.	Mild sedation, increased appetite, fatigue, dizziness, drowsiness, tremor, and constipation	McDougle et al., 1998b; McCracken et al., 2002; Hara et al., 2017
	Aripiprazole	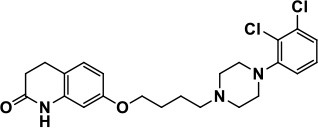	Reduces autistic symptoms in children such as irritability, stereotypy, and hyperactivity	Fatigue, vomiting, weight gain, tremor, and extrapyramidal symptoms	Marcus et al., 2009; Owen et al., 2009; Hirsch and Pringsheim, 2016
**Selective Serotonin Reuptake inhibitor (SSRI)**	Fluoxetine	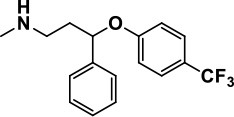	Reduces stereotyped and repetitive behavior in children and adolescents with ASD.	Hypomania. agitation, and hyperactivity	Fatemi et al., 1998; DeLong et al., 2002; Hollander et al., 2005; Hendriksen et al., 2016
	Fluvoxamine	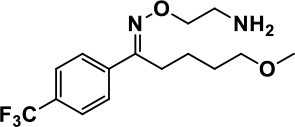	Improves compulsive repetitive behaviors, aggression	Irritability, and increase risk of suicidal ideas	McDougle et al., 1996; Martin et al., 2003; Brown et al., 2017; Howes et al., 2018; Lee et al., 2018
	Sertraline	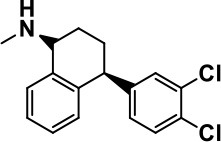	All SSRIs possess similar effects to fluoxetine and fluvoxamine. Sertraline shows improvements in repetitive and disruptive behavior in adults with ASD, paroxetine reduced aggression, and escitalopram shows improvements in irritability, stereotypy, hyperactivity and inappropriate speech.		Hellings et al., 1996; McDougle et al., 1998a; AlOlaby et al., 2017
	Paroxetine	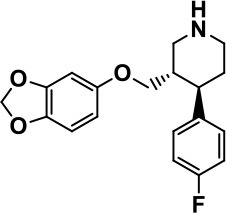			Davanzo et al., 1998; Hellings et al., 2015
	Escitalopram	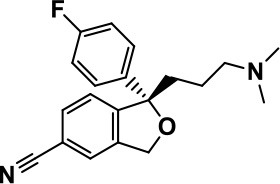			Owley et al., 2005; Brown et al., 2017; Viktorin et al., 2017
	Venlafaxine	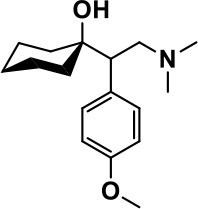	Improves restricted behavior and interest, social and communication deficits, and hyperactivity	Irritability, and increase risk of suicidal idea	Carminati et al., 2006, 2016
**Tricyclic Antidepressant**	Nortriptyline	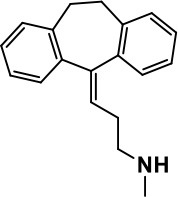	Improves hyperactivity and aggressiveness in autistic children	Sedation, increase in aggression, irritability and hyperactivity	Kurtis, 1966; Campbell et al., 1971; Hong et al., 2017
	Clomipramine	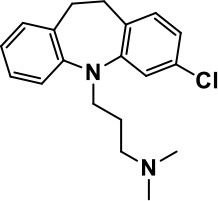	Improves anger and compulsive and ritualized behavior		Gordon et al., 1993; Sanchez et al., 1996; Hong et al., 2017
**Anticonvulsants**	Lamotrigine	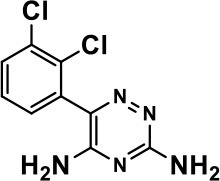	Improves overall autistic symptoms	Life-threatening skin reactions, including Stevens-Johnson syndrome, drug reaction with eosinophilia and systemic symptoms, and toxic epidermal necrolysis	Uvebrant and Bauziene, 1994; Jobski et al., 2017
	Valproic acid	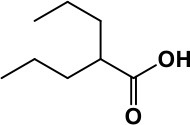	Improves receptive language, affective instability, aggression, and social skills	Irritability, weight gain, anxiety	Anagnostou et al., 2006; Hollander et al., 2006, 2010; Jobski et al., 2017
	Levetiracetam	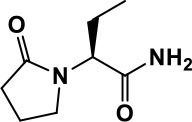	Decreases symptoms such as hyperactivity, impulsivity, aggression, and affective lability	CNS effects such as somnolence, decreased energy, headache, dizziness, mood swings and coordination difficulties	Rugino and Samsock, 2002; Jobski et al., 2017
**Glutamate antagonist**	Amantadine	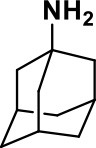	Improves hyperactivity and speech disturbance	Nervousness, anxiety, agitation, insomnia, difficulty in concentrating, and exacerbations of pre-existing seizure disorders and psychiatric symptoms in patients with schizophrenia or Parkinson's disease.	King et al., 2001; Naaijen et al., 2017
	Memantine	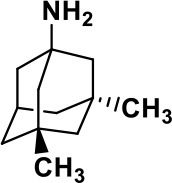	Improves memory, hyperactivity, irritability, social behavior and communication, and self-stimulatory behavior	Few autistic individuals experienced worsening of autistic symptoms	Owley et al., 2006; Chez et al., 2007; Naaijen et al., 2017; Vorstman et al., 2017
**Acetylcholinesterase inhibitors**	Rivastigmine	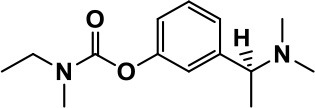	Improves overall autistic behavior	Nausea, diarrhea, hyperactivity, and irritability	Vorstman et al., 2017
	Donepezil	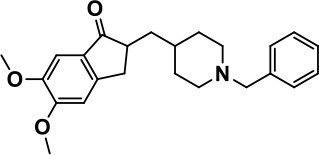	Improves irritability and hyperactivity	Nausea and vomiting, decreased appetite and weight loss	Hardan and Handen, 2002; Vorstman et al., 2017
	Galantamine	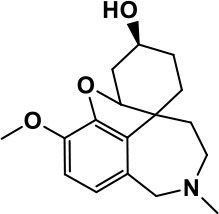	Improves several autistic symptoms in children such as irritability, hyperactivity, social interaction deficits, inappropriate speech, loss of attention, and anger		Niederhofer et al., 2002; Nicolson et al., 2006; Vorstman et al., 2017
**Psycho-stimulant**	Methylphenidate	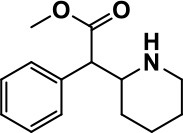	Improves several autistic behavioral symptoms in children and adolescents such as hyperactivity, impulsivity, attention, social communication, and self-regulation	Anorexia, aggression, and insomnia	Handen et al., 2000; Di Martino et al., 2004; Jahromi et al., 2009; Kim et al., 2017
**Adrenergic** α_2_ **receptor agonists**	Clonidine	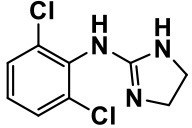	Improves hyperactivity, mood fluctuation, aggressiveness and agitation, sleeping pattern and night time awakenings	Sedation, dry mouth, and hypotension	Fankhauser et al., 1992; Ming et al., 2008; Nash and Carter, 2016
	Guanfacine	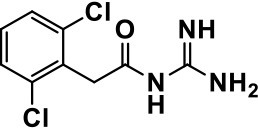	Improves attention, hyperactivity, and tics	Insomnia, fatigue, blurred vision, mood instability, sedation, constipation, irritability, and aggression.	Posey et al., 2004; Scahill et al., 2006; Boellner et al., 2007; Nash and Carter, 2016
**Opiate antagonist**	Naltrexone	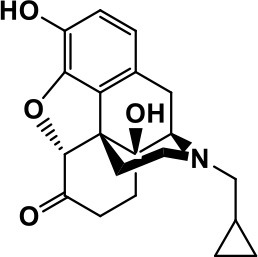	Improves, hyperactivity, irritability, self- injuries. However, ineffective in social deficits.	Gastrointestinal complaints such as diarrhea and abdominal cramping.	Panksepp and Lensing, 1991; Bouvard et al., 1995; Kolmen et al., 1995; Elchaar et al., 2006; Clifford et al., 2007; Nash and Carter, 2016

*Data were obtained from https://clinicaltrials.gov/ last accessed 2017 August 20th*.

### Atypical antipsychotics

Clozapine belongs to the class of atypical antipsychotics because it shows capability of binding to 5-HT and DA receptors (Zuddas et al., 1996; Chen et al., 2001; Gobbi and Pulvirenti, 2001). It is an atypical antipsychotic medication mainly used for SCH that does not improve following the use of other antipsychotic medications. In patients with SCH and schizoaffective disorder it may decrease the rate of suicidal behavior. It is possibly more effective than typical antipsychotics and in patients who are resistant to other medications. Clozapine was found to improve hyperactivity and aggression in ASD children, adolescents and adults, but has a limited clinical use because of it's hematological safety profile a potential of lowering the seizure threshold in epilepsy patients, necessitating monitoring procedures of patients taking this medication (Zuddas et al., 1996; Chen et al., 2001; Gobbi and Pulvirenti, 2001). The antipsychotic medication with risperidone is mainly approached in ASD patients with SCH, bipolar disorder, and/or irritability symptoms, as this drug has revealed to be better than placebo in treating irritability, repetitive behavior, aggression, anxiety, depression and nervousness (McDougle et al., 1998b; McCracken et al., 2002). Moreover, risperidone has shown a neuroprotective effect and has enhanced the antioxidant and neuroprotective activity of astroglial cells in brain disorders such as ASD without clinical evidence of extrapyramidal side effects or seizures except mild sedative effects (McDougle et al., 1998b; McCracken et al., 2002). However, other side effects with use of risperidone were reported to include increased appetite, fatigue, dizziness and drowsiness (Table 1; McDougle et al., 1998b; McCracken et al., 2002). Aripiprazole is another atypical antipsychotic primarily recommended for the treatment of SCH and bipolar disorder. Other uses include an add-on treatment for major depressive disorder, tic disorders, and irritability associated with ASD, as it shows a different mechanism of action from those of the other atypical antipsychotics (e.g., clozapine, and risperidone) by acting rather as a partial agonist on the D2Rs and 5-HT1A receptors (Marcus et al., 2009; Owen et al., 2009; Hirsch and Pringsheim, 2016). However, it displays an antagonist profile at 5-HT2A and 5-HT7 receptors and acts as a partial agonist at the 5-HT2C receptor, both with high affinity (Marcus et al., 2009; Owen et al., 2009; Hirsch and Pringsheim, 2016). The latter action may be the reason for the minimal weight gain observed during the course of therapy with aripiprazole.

### Neurotransmitter reuptake inhibitors

The class of neurotransmitter reuptake inhibitors, e.g., fluoxetine (selective serotonin reuptake inhibitor, SSRI) has shown numerous prospective therapeutic benefits, including decreases in rituals, stereotyped and repetitive monotonous behaviors in ASD children and adolescents (Fatemi et al., 1998; DeLong et al., 2002; Hollander et al., 2005). Fluoxetine belongs to the class of SSRI which does not significantly inhibit norepinephrine and dopamine reuptake at therapeutic doses (Fatemi et al., 1998; DeLong et al., 2002; Hollander et al., 2005). Fluoxetine was found to produce some adverse effects including disinhibition, hypomania, agitation, and hyperactivity (Fatemi et al., 1998; DeLong et al., 2002; Hollander et al., 2005). Fluvoxamine is a drug which functions as a SSRI and σ1 receptor agonist (Fatemi et al., 1998; DeLong et al., 2002; Hollander et al., 2005). Fluvoxamine is used mainly for the treatment of obsessive-compulsive disorder, and is also used to treat major depressive disorder and anxiety disorders such as panic disorder and post-traumatic stress disorder. Notably, fluvoxamine is approved to treat social anxiety disorder (Fatemi et al., 1998; DeLong et al., 2002; Hollander et al., 2005). Fluvoxamine has also shown similar potential effects as fluoxetine against ASD (Fatemi et al., 1998; DeLong et al., 2002; Hollander et al., 2005). In a clinical trial, fluvoxamine was found to be well tolerated in ASD adults and it has improved compulsive as well as repetitive behaviors and aggression (Fatemi et al., 1998; DeLong et al., 2002; Hollander et al., 2005). Other SSRIs, including sertraline, paroxetine and escitalopram, showed almost the same potential benefits and adverse effects as compared to fluoxetine and fluvoxamine (Hellings et al., 1996; McDougle et al., 1998a). Venlafaxine is another SSRI which has, also, shown improvements of restricted behaviors, decreased interests, social deficits, hyperactivity and communication problems in individuals with ASD, (Table 1; Hellings et al., 1996; McDougle et al., 1998a).

### Tricyclic antidepressants

The second-generation tricyclic antidepressant nortriptyline is used in the therapeutic management of major depression and childhood nocturnal enuresis (bedwetting), chronic fatigue syndrome, chronic pain and migraine, and labile affect in some neurological brain disorders (Kurtis, 1966; Campbell et al., 1971). Clomipramine is another tricyclic antidepressant used for the treatment of obsessive compulsive disorder, panic disorder, major depressive disorder, and chronic pain (Gordon et al., 1993; Sanchez et al., 1996). Interestingly, nortriptyline has been described to be effective in children with ASD as it improved the hyperactivity, aggressiveness, and ritualized behavior, while imipramine was not well tolerated in ASD children (Kurtis, 1966; Campbell et al., 1971). In a previous clinical trial, 58% of ASD subjects have found clomipramine to be superior to placebo and the antidepressant desipramine in improving ASD symptoms, anger, and compulsive and ritualized behaviors (Gordon et al., 1993). In another clinical study, clomipramine has caused several adverse effects such as sedation and worsening of behaviors like aggression, irritability, and hyperactivity (Sanchez et al., 1996; Table 1).

### Anticonvulsants

Lamotrigine, a member of the sodium channel blocking class of anticonvulsants clinically used in the treatment of children diagnosed with epilepsy, decreased symptoms in approximately 62% of the ASD individuals, and no considerable change among placebo-treated and lamotrigine-treated patients was observed in a study comprising 35 patients diagnosed with ASD (Uvebrant and Bauziene, 1994). On the contrary, VPA with its anticonvulsant effect recognized based on its blockade of voltage-dependent sodium channels and increased GABA levels in the brain has shown -as an orphan drug- valuable effects in improving various symptoms and psychiatric comorbidities, e.g., receptive language, affective instability, and aggression, without appreciable clinical effects on core symptoms of ASD (Uvebrant and Bauziene, 1994; Jobski et al., 2017). Notably, VPA has been reported to be an inhibitor for histone deacetylase (HDAC), an enzyme which plays –together with other HDACs- an essential regulating role in gene transcription and phenotypic differentiation (Hsieh and Gage, 2004, 2005; Balasubramaniyan et al., 2006; Jessberger et al., 2007; Chomiak et al., 2013). Accordingly, numerous studies reported that specific expression forms of HDAC1 and HDAC2, which are categorized as class I of HDACs, in the murine brain are existent at various developmental ages with HDAC1 expressed in neural stem cells/progenitors and glia, and HDAC2 being upregulated in postmitotic neuroblasts and various but not in fully differentiated glia (Chomiak et al., 2013). Therefore, modulation of HDAC in diverse cell types and at several maturational time points may possibly lead to the observation of intensely diverse clinical outcomes and may explain why HDAC inhibition (for instance with VPA) in adulthood leads to improvement of ASD-like features in animals exposed prenatally to VPA (Chomiak et al., 2013). In previous studies, in utero exposure to VPA in mice induced ASD-like behavioral social interaction deficits, anxiety and spatial learning incapacity (Kataoka et al., 2013). However, all these behavioral impairments were ameliorated following chronic (5-week) treatment with VPA (30 mg/kg/d, i.p.) (Kataoka et al., 2013; Takuma et al., 2014), suggesting that dose (300-600/kg mg for acute induction of ASD or 30 mg/kg for chronic treatment goals) or time point (prenatal or 8-week old animals) of using VPA determines whether it is ASD-inducing or palliating cognitive dysfunction (Takuma et al., 2014). VPA-induced ASD in rodents will be discussed in more details under section Non-genetic Animal Models of ASD. Moreover, the anticonvulsant drug levetiracetam was found to be valuable in decreasing hyperactivity, impulsivity, aggression, and emotional lability (Rugino and Samsock, 2002) (Table 1). These clinical observations for several antiepileptic drugs demonstrate that the prevalence of psychopharmacotherapy and polypharmacy in ASD patients is considerable, which is probably due to the treatment of non-core ASD symptoms and psychiatric comorbidities, despite a lack of pharmacological treatment options for ASD core symptoms.

### Glutamate antagonists

Levels of glutamate have been found to be excessively increased in post-mortem brain samples of some ASD individuals (Owley et al., 2006; Chez et al., 2007). Numerous studies have publicized the effectiveness of several glutamate antagonists, e.g., amantadine and memantine, in ASD patients (Owley et al., 2006; Chez et al., 2007). In a controlled clinical trial, amantadine showed improving effects on hyperactive behavior and inappropriate speech in ASD children (Owley et al., 2006; Chez et al., 2007). Also, the clinical use of memantine in the treatment of ASD individuals has shown therapeutic progresses in regard to memory, hyperactivity, irritability, language, social behavior and self-stimulatory behavior (Owley et al., 2006; Chez et al., 2007; Table 1).

### Acetylcholinesterase inhibitors

Dysfunction of brain cholinergic neurotransmission has been described in several patients diagnosed with ASD (Perry et al., 2001; Hardan and Handen, 2002; Niederhofer et al., 2002; Nicolson et al., 2006). Therefore, acetylcholinesterase inhibitors, e.g., rivastigmine, donepezil, and galantamine, have in many studies been investigated for the use in ASD children (Hardan and Handen, 2002; Niederhofer et al., 2002; Nicolson et al., 2006). Interestingly, the clinical application of rivastigmine in ASD children significantly relieved overall ASD behaviors, however, several adverse effects including nausea, diarrhea, hyperactivity and irritability were reported (Hardan and Handen, 2002; Niederhofer et al., 2002; Nicolson et al., 2006). Among acetylcholinesterase inhibitors, donepezil has shown capability to improve irritability and hyperactivity of ASD children (Hardan and Handen, 2002; Niederhofer et al., 2002; Nicolson et al., 2006). Moreover, galantamine produced substantial improvements in hyperactivity, irritability, social withdrawal, inappropriate speech, attention deficiency, and reduction in anger in children diagnosed with ASD (Hardan and Handen, 2002; Niederhofer et al., 2002; Nicolson et al., 2006; Table 1). These improvements observed for several acetylcholine esterase inhibitors strongly support the hypothesis that enhancing the cholinergic neurotransmission in ASD results in positive therapeutic effects.

### Psychostimulants

Methylphenidate, the most commonly known CNS stimulant widely used in the therapeutic management of attention deficit hyperactivity disorder (ADHD) and narcolepsy, is commonly indicated for ASD children and adolescents (Handen et al., 2000; Di Martino et al., 2004; Jahromi et al., 2009; Kim et al., 2017). Methylphenidate mainly acts as a norepinephrine-dopamine reuptake inhibitor (NDRI). In numerous controlled studies, methylphenidate palliated several behavioral ASD features including impulsivity, attention deficiency, and hyperactivity, but it correspondingly exhibited some initial adverse effects such as aggression, anorexia, and increased wakefulness (insomnia) (Handen et al., 2000; Di Martino et al., 2004; Jahromi et al., 2009; Kim et al., 2017; Table 1).

### Adrenergic alpha (α)_2_ receptor agonists

Oral or transdermal administration of selective centrally acting α_2_ adrenergic agonist, e.g., clonidine, have revealed to improve mood instability, hyperactive behavior, aggressiveness and nervousness in ASD individuals (Fankhauser et al., 1992; Ming et al., 2008). Clonidine is a drug used to treat high blood pressure, ADHD, anxiety disorders, tic disorders, withdrawal (from either alcohol, opioids, or smoking), migraine, and certain pain conditions, with largely tolerable adverse effects (Fankhauser et al., 1992; Ming et al., 2008). Also, previous clinical trials carried out with clonidine in ASD subjects delivered evidences for the clinical effectiveness and safety profile in ASD and related brain disorders (Fankhauser et al., 1992; Ming et al., 2008). Moreover, a retrospective study revealed that the use of guanfacine, a selective α_2_ adrenergic receptor agonist used in the treatment of ADHD, anxiety, and hypertension, was accompanied with enhancements in insomnia, attention deficiency, hyperactivity, and tics (Posey et al., 2004; Scahill et al., 2006; Boellner et al., 2007). However, the most common adverse effects observed with guanfacine were mood alteration fatigue, blurred vision, and headache (Posey et al., 2004; Scahill et al., 2006; Boellner et al., 2007; Table 1).

### Opiate antagonists

Based on the reputed role of endogenous opioids such as β-endorphin and encephalin in the regulation of social behavior, the opiate antagonist naltrexone has been assessed in ASD (Panksepp and Lensing, 1991; Bouvard et al., 1995; Kolmen et al., 1995; Elchaar et al., 2006; Clifford et al., 2007). The results observed in numerous studies for naltrexone showed that it might be able to treat behavioral aberrations perceived in ASD patients and induced by dysfunction of the brain opioid system (Panksepp and Lensing, 1991; Bouvard et al., 1995; Kolmen et al., 1995; Elchaar et al., 2006; Clifford et al., 2007). Moreover, numerous studies revealed the significant improvements of various behavioral symptoms obtained with the use of naltrexone in ASD children (Panksepp and Lensing, 1991; Bouvard et al., 1995; Kolmen et al., 1995; Elchaar et al., 2006; Clifford et al., 2007). Furthermore, these studies reported that naltrexone treatment provided substantial enhancements in self-injurious behavior, hyperactivity, social withdrawal, agitation and irritability in ASD patients (Panksepp and Lensing, 1991; Bouvard et al., 1995; Kolmen et al., 1995; Elchaar et al., 2006; Clifford et al., 2007; Table 1).

The aforementioned described drugs act on different targets to therapeutically manage the behavioral as well as psychiatric symptoms of ASD. However, several brain regions are altered in ASD individuals, resulting in loss of neuronal function, and behavioral and sensory impairments, including inattention, hyperactivity, mood fluctuations, aggressiveness, agitation, social deficits and repetitive and restricted behavior. In the brain, the plasticity of brain tissue, nerve tangling and imbalanced production of several neurotransmitters are all implicated on evolution in ASD individuals. Apart from environmental factors, other pathological conditions such as immunological problems, chronic neuroinflammation, oxidative stress, mitochondrial dysfunction are involved in etiopathogenesis of ASD (Kumar et al., 2012).

Up until now, there is no approved drug existing in the market which is specific for treating symptoms associated with ASD, however, preclinical and clinical research and development are in progress to find new therapeutic entities. Currently, there are several candidates that successfully passed different clinical phases of drug development, with different pharmacological targets to palliate ASD behavioral and neurological symptoms (Table 2, Figure 3). Interestingly, 10% of these clinical candidates are currently in phase 1, 46% have already progressed to phase 2, 27% advanced to phase 3, and 15% reached to phase 4 of clinical development, whereas 2% are currently of preclinical interest (Ruhela et al., 2015; Table 2, Figure 3).

**Table 2:** Drugs currently in phase 1–4 of clinical development for neurobehavioral manifestations, neurodevelopmental, and autistic disorders.

**Name**	**Class**	**Clinical indication/condition (No. of trials in phase 2)**	**Clinical indication/condition (No. of trials in phase 3)**	**Clinical indication/condition (No. of trials in phase 4)**
Arbaclofen	Anticonvulsant	Autistic disorder (3) Neurobehavioral Manifestations (2) Neurodevelopmental Disorders (3)	Autistic disorder (2) Neurobehavioral Manifestations (3) Neurodevelopmental Disorders (1)	
Aripiprazole	Antipsychotic drug	Autistic Disorder (5) Neurobehavioral Manifestations (1) Neurodevelopmental Disorders (6)	Autistic Disorder (7) Neurobehavioral Manifestations (1) Neurocognitive Disorders (6) Neurodegenerative disease (11) Neurodevelopmental Disorders (13)	Autistic Disorder (5) Neurocognitive Disorders (3) Neurodegenerative disease (3) Neurodevelopmental Disorders (11)
Atomoxetine	Selective norepinephrine transporter	Neurobehavioral Manifestations (4) Neurocognitive Disorders (5) Neurodegenerative disease (4) Neurodevelopmental Disorders (14)	Autistic Disorder (1) Neurocognitive Disorders (1) Neurodegenerative disease (1) Neurodevelopmental Disorders (29)	Autistic Disorder (3) Neurobehavioral Manifestations (5) Neurocognitive Disorders (1) Neurodegenerative disease (2) Neurodevelopmental Disorders (48)
Baclofen	Central muscle relaxant	Autistic Disorder (2) Neurobehavioral Manifestations (1) Neurodegenerative Diseases (2) Neurologic Manifestations (9)	Neurobehavioral Manifestations (1) Neurodegenerative Diseases (1)	
Bumetanide	Diuretic action	Autistic Disorder (2)	Autistic Disorder (1)	Neurodevelopmental Disorders (1)
Buspirone	Anxiolytic	Autistic disorder (3) Neurodegenerative Diseases (1) Neurodevelopmental Disorders (4)	Autistic disorder (1) Neurocognitive Disorders (1) Neurodegenerative Diseases (1)	
Citalopram	Antidepressant	Autistic Disorder (1) Neurocognitive Disorders (4) Neurodegenerative Diseases (2) Neurodevelopmental Disorders (2)	Neurobehavioral Manifestations (3) Neurocognitive Disorders (7) Neurodegenerative Diseases (7)	Neurobehavioral Manifestations (3) Neurocognitive Disorders (4) Neurodegenerative Diseases (6)
CX516		Autistic Disorder (1) Neurocognitive Disorders (2) Neurodegenerative Diseases (1)		
D-Cyclocerine	NMDA modulator			
Dimercaptosuccinic acid	Antidote for lead, mercury, and arsenic poisoning	Autistic Disorder (1)		
Donepezil	Acetylcholinesterase inhibitor	Autistic Disorder (4) Neurobehavioral Manifestations (7) Neurocognitive Disorders (62) Neurodegenerative Diseases (57)	Neurobehavioral Manifestations (5) Neurocognitive Disorders (41) Neurodegenerative Diseases (33)	Autistic disorder (1) Neurobehavioral Manifestations (6) Neurocognitive Disorders (49) Neurodegenerative Diseases (37) Neurodevelopmental Disorders (1)
Fluoxetine	SSRI	Autistic disorder (3) Neurodegenerative disease (1)	Autistic disorder (3) Neurocognitive Disorders (1)	Neurodegenerative Diseases (1) Neurodevelopmental Disorders (1)
Fluvoxamine	SSRI		Autistic disorder (1) Neurocognitive Disorders (1) Neurodevelopmental Disorders (2)	Neurodegenerative Diseases (1)
Galantamine	Cholinesterase inhibitor	Neurobehavioral Manifestations (2) Neurocognitive Disorders (12) Neurodegenerative disease (7)	Autistic Disorder (1) Neurocognitive Disorders (28) Neurodegenerative disease (21) Neurodevelopmental Disorders (1)	Neurobehavioral Manifestations (3) Neurocognitive Disorders (22) Neurodegenerative disease (21) Neurodevelopmental Disorders (2)
Lenalidomide	Anti-inflammatory	Autistic Disorder (1) Neurodegenerative Diseases (1)		
Levetiracetam	Anticonvulsant	Neurobehavioral Manifestations (1) Neurocognitive Disorders (2) Neurodegenerative disease (5)	Neurocognitive Disorders (1)	Neurobehavioral Manifestations (2) Neurocognitive Disorders (1) Neurodegenerative disease (3) Neurodevelopmental Disorders (3)
				
Methyl cobalamin	Vitamin	Autistic disorder (2) Neurocognitive Disorders (1) Neurodegenerative Diseases (4)	Autistic disorder (2) Neurocognitive Disorders (1) Neurodegenerative Diseases (5) Neurodevelopmental Disorders (2)	Neurocognitive Disorders (4) Neurodegenerative Diseases (1)
Minocycline	Antibiotic	Autistic Disorder (1) Neurobehavioral Manifestations (3) Neurocognitive Disorders (5) Neurodegenerative disease (5)	Neurobehavioral Manifestations (3) Neurocognitive Disorders (2) Neurodegenerative disease (2)	Autistic Disorder (1) Neurobehavioral Manifestations (1)
*N*-Acetyl cysteine	Antioxidant	Autistic disorder (4) Neurobehavioral Manifestations (4) Neurocognitive Disorders (4) Neurodegenerative Diseases (7) Neurodevelopmental Disorders (5)	Neurobehavioral Manifestations (1) Neurodegenerative Diseases (2)	Neurocognitive Disorders (1) Neurodegenerative Diseases (1)
Naltrexone	Opioid receptor antagonist	Autistic Disorder (1) Neurobehavioral Manifestations (2) Neurocognitive Disorders (1) Neurodegenerative disease (1)	Neurobehavioral Manifestations (1) Neurodevelopmental Disorders (1)	Neurodegenerative disease (1) Neurodevelopmental Disorders (3)
Olanzapine	Atypical antipsychotic	Autistic Disorder (2) Neurocognitive Disorders (1) Neurodegenerative Diseases (1) Neurodevelopmental Disorders (2)	Autistic Disorder (1) Neurobehavioral Manifestations (2) Neurocognitive Disorders (3) Neurodegenerative Diseases (2) Neurodevelopmental Disorders (2)	Neurobehavioral Manifestations (2) Neurocognitive Disorders (6) Neurodegenerative Diseases (2) Neurodevelopmental Disorders (5)
Oralgam	human immunoglobulin	Autistic Disorder (1) Child Development Disorders, Pervasive (1) Neurodevelopmental Disorders (1)		
Oxytocin hormone	Hormone	Autistic Disorder (5)		
Paliperidone ER	Atypical antipsychotic		Autistic Disorder (1) Neurocognitive Disorders (1) Neurodevelopmental Disorders (1)	Neurobehavioral Manifestations (1) Neurocognitive Disorders (1)
Pioglitazone	Antihyperglycemic	Autistic Disorder (1) Neurocognitive Disorders (2) Neurodegenerative Diseases (4)	Neurocognitive Disorders (2) Neurodegenerative Diseases (3)	
RG 1068	Synthetic human secretin		Autistic Disorder (2)	
Riluzole	Sodium channel blocker	Autistic Disorder (3) Neurocognitive Disorders (2) Neurodegenerative Disorders (34)	Autistic disorder (1) Neurocognitive Disorders (1) Neurodegenerative Diseases (11)	Neurobehavioral Manifestations (2) Neurodegenerative Diseases (3) Neurodevelopmental Disorders (1)
Risperidone	Atypical antipsychotic drug	Autistic Disorder (4) Neurocognitive Disorders (3) Neurodegenerative disease (1) Neurodevelopmental Disorders (7)	Autistic Disorder (6) Neurobehavioral Manifestations (4) Neurocognitive Disorders (6) Neurodegenerative disease (5) Neurodevelopmental Disorders (12)	Autistic Disorder (4) Neurobehavioral Manifestations (8) Neurocognitive Disorders (11) Neurodegenerative disease (5) Neurodevelopmental Disorders (10)
Saproterin	Enzymatic cofactor			
Sertraline	SSRI	Autistic Disorder (2) Neurobehavioral Manifestations (2) Neurocognitive Disorders (1) Neurodegenerative disease (2) Neurodevelopmental Disorders (2)	Autistic Disorder (1) Neurobehavioral Manifestations (1) Neurocognitive Disorders (2) Neurodegenerative disease (1) Neurodevelopmental Disorders (2)	Neurocognitive Disorders (2) Neurodegenerative disease (1) Neurodevelopmental Disorders (2)
Valproic acid	Anticonvulsant	Autistic Disorder (3) Neurobehavioral Manifestations (2) Neurocognitive disorders (1) Neurodegenerative Diseases (7)	Autistic disorder (1) Neurobehavioral Manifestations (2) Neurocognitive Disorders (3) Neurodegenerative Diseases (3) Neurodevelopmental Disorders (1)	Autistic disorder (1) Neurobehavioral Manifestations (3) Neurocognitive Disorders (3) Neurodegenerative Diseases (1) Neurodevelopmental Disorders (6)
Valproic acid + sodium valproate	Anticonvulsant		Neurobehavioral Manifestations (1) Neurocognitive Disorders (1)	Autistic Disorder (1) Neurobehavioral Manifestations (1) Neurocognitive Disorders (1)
				
Ziprasidone	Atypical antipsychotic	Autistic Disorder (1) Neurobehavioral Manifestations (1) Neurocognitive Disorders (2) Neurodevelopmental Disorders (2)	Autistic disorder (1) Neurobehavioral Manifestations (3) Neurocognitive Disorders (3)	Neurobehavioral Manifestations (2) Neurocognitive Disorders (2) Neurodevelopmental Disorders (2)

## Animal models of ASD

For the understanding of the etiology and pathogenesis of any human disease, e.g., ASD, experimental rodent models are of substantial meaning. Modelling of human neuropsychiatric diseases with animals is very challenging due to the multifaceted nature of these disorders, combined with the absence of effective diagnostic biomarkers and objective tests for accurate diagnosis (Nestler and Hyman, 2010). Moreover, neuropsychiatric diseases, such as ASD, are multifactorial with genetic, medical and neurodevelopmental conditions associated with the disease (Hoffman et al., 2017; Lyall et al., 2017; Xie et al., 2017). Generally, there are two types of rodent models for ASD, namely the genetic and non-genetic animal models. Accordingly, ASD genetic animal models are highly applicable when they reproduce the ASD features that are existent in an individual human genetic disease such as tuberous sclerosis and fragile X syndrome. Therefore, the ASD genetic models induced in mice should be established on an identified genetic cause of a disease, reflect key aspects of the human symptoms and respond to pharmacotherapies that are operational in the human diseases (Chadman et al., 2009).

### Genetic animal models of ASD

A considerable advancement in detecting the genetic basis of several human diseases has been achieved by geneticists. These achievements include Alzheimer's disease, Huntington's disease, and some forms of breast cancer. However, more difficulties were associated with the detection of genetic factors for multifaceted diseases such as SCH, diabetes, bipolar disorder, and also ASD (Rao et al., 2013; Wei et al., 2015; Fregeac et al., 2016; Hu et al., 2017). In the case of ASD, extensive research efforts elucidated underlying mechanisms of the disorder and concluded that these mechanisms might include dysfunctions of neuronal microRNAs (miRNAs), which are regulators of gene expression, and which have gained much attention in pathophysiology of various psychiatric illnesses (Andolina et al., 2017). Accordingly, about half of all identified miRNAs in humans are expressed in the CNS and exhibit modulatory functions crucial for numerous biological processes associated to the development of the CNS (Hu et al., 2017). Consequently, disruptions in miRNA biogenesis and miRNA-target interaction have been linked with CNS diseases, including ASD (Hu et al., 2017). Moreover, three miRNA (miRNA-7, miRNA-9, and miRNA-106b) were found to be associated with neurodegenerative diseases and only one, namely miRNA-9, with intellectual disability (Doxakis, 2010; Wang et al., 2010; Xu et al., 2011; Hu et al., 2017). Furthermore, it was recognized that the intellectual disability in Down syndrome which is a neurodevelopmental disorder, was caused by an extra-copy of chromosome 21 (Lejeune et al., 1959). However, it is now being firmly established that deletions in the chromosome 21 are commonly discovered in some patients with intellectual disability including ASD (Hogart et al., 2010). Similarly, increased risk of delayed development, intellectual disability and neurological as well as psychiatric problems associated with ASD, SCH, epilepsy and hypotonia was found to be the result of micro deletions in the long (q) arm of the chromosome in a region designated q21.1 (Knight et al., 1999; Ravnan et al., 2006; Mefford et al., 2009). Moreover, chromosome 15 has been described to be as one of seven several chromosomes enriched in segmental low copy repeats or duplicons (Bailey et al., 2002). These duplicons were revealed to strongly contribute to a mechanism in which low copy repeats facilitated misalignment during meiosis I, leading to unequal recombination of nonallelic homologes and developing a sequence of common cut-off point along the 15q11.2-q13 region (Robinson et al., 1993a,b,c; Christian et al., 1999). Previous reports suggested, also, several cases of ASD with cytogenetic abnormalities in the 15q11–13 region (Cook et al., 1998), and the frequency of cytogenetic abnormalities in this region was found to be around 0-3% in ASD, and in fact, the most frequent cytogenetic abnormality in this population (Lord et al., 2000). Moreover, it has been reported that the paternal inheritance of a cytogenetical abnormality leads to a normal phenotype; maternal inheritance leads to autism or atypical autism (Tabet et al., 2015). Furthermore, previous efforts revealed that 16p13.1 micro duplication syndrome is caused by interstitial duplications encompassing 16p13.1, which is a risk factor for a range of neuropsychiatric disorders, including ASD, intellectual disability, SCH, and ADHD (Ullmann et al., 2007; Mefford et al., 2009; Ramalingam et al., 2011; Grozeva et al., 2012). There are, also, monogenic diseases that have been associated with ASD and display its characteristic features: tuberous sclerosis, fragile X syndrome, Rett syndrome and neurofibromatosis 1 (Wetmore and Garner, 2010; Hulbert and Jiang, 2016). Accordingly, single gene mutation gave rise to several human syndromes leading to an increase in the risk for developing ASD. Moreover, autistic traits are also connected to copy-number variants that lead to inheritance of maternal 15q11–13 duplication, resulting in Prader-Willi syndrome (Ogata et al., 2014). Furthermore, the developed strategies for the identification of genetic variants has led to the description of novel syndromic forms of ASD and facilitated the relationship between genetic traits and phenotype. Consequently, these genetic dissimilarities identified so far, along with the newly developed strategies for genetic engineering, improved the expansion of genetic animal models for ASD. Accordingly, mice are the major animal model for ASD due to their genetic manipulability and their talent to show behavioral deficits associated with ASD features (Chen et al., 2015). Moreover, construct as well as face validity of mice ASD animal models together with the availability of well-established techniques to manipulate their genome and study brain function at numerous levels of analysis constitute to the overall validity of mice model (Hulbert and Jiang, 2016). Also, mice as mammals are genetically and biologically similar to humans, however, their rapid reproduction and accelerated development allow for the testing of large numbers of animals at a relatively low cost (Hulbert and Jiang, 2016). Accordingly, animal models for genes of a syndromic disorder were commonly found to present autistic traits including Fragile X (FMR1) with social interaction deficits, hyperactivity, and cognitive impairments (Willem Verhoeven, 2011; Bhattacharya et al., 2012; Ronesi et al., 2012; Hulbert and Jiang, 2016; Hu et al., 2017), Rett syndrome and MECP2 mutations with the resulted repetitive and stereotypic/restricted behaviors, abnormal gait and reduced anxiety, decreased pain and normal olfactory discrimination (Chao et al., 2010; Samaco et al., 2013), tuberous sclerosis TSC1 or TSC2 with social interactions deficits and repetitive/restricted behavior or interest (Chao et al., 2010; Willem Verhoeven, 2011), Timothy syndrome (TS) CACNA1C with impairments in social interactions, repetitive/stereotypic behaviors, and increased fear conditioning (Ergaz et al., 2016), Phelan-McDermid syndrome with restricted interest as well as cognitive and motor deficits (Giza et al., 2010; Ergaz et al., 2016), PTEN mutations, cortical dysplasia focal epilepsy with deficits in regard to social interactions, restricted interest, sensory sensitivity, elevated anxiety, and seizures (Scott-Van Zeeland et al., 2010) (Cook and Scherer, 2008; LaSalle et al., 2015; Piochon et al., 2015) as behavioral features of the above described syndromes which are associated with the ASD (Table 3). Numerous inbred mouse strains show face validity as ASD models in addition to the genetically modified animal models of ASD, as such inbred strains display robust and well-replicated social deficits and repetitive behaviors (Kazdoba et al., 2016). These inbred strains are found to display idiopathic autism, as their ASD-relevant behaviors are not caused by known genetic mutations (Kazdoba et al., 2016). For instance and while assessing sociability, inbred strains like A/J, BALB/cByJ (BALB), BTBR T+Itpr3tf/J (BTBR), C58/J (C58), and 129S1/SvImJ mice showed lack of sociability, as compared to inbred mouse strains with high sociability, such as C57BL/6J (B6) and FVB/NJ mice (Brodkin, 2007; Moy et al., 2007, 2008; McFarlane et al., 2008; Kazdoba et al., 2016; Hu et al., 2017). Moreover, it has been shown that several mouse strains, e.g. BTBR and C58, also demonstrate explicit motoric stereotypies or repetitive behaviors, such as jumping, digging, and high levels of self-grooming and marble burying (Pasciuto et al., 2015; Kazdoba et al., 2016).

**Table 3:** Most relevant ASD mouse models induced by targeting ASD-associated genes.

**Mouse model**	**Associated syndrome**	**Targeted mutant gene (Chromosome)**	**Observed behavioral symptoms**	**References**
Fmr1 KO mice	Fragile X syndrome	FMR1 (Xq27)	Social interaction deficits, hyperactivity, cognitive impairments.	Willem Verhoeven, 2011; Bhattacharya et al., 2012; Ronesi et al., 2012; Hulbert and Jiang, 2016; Hu et al., 2017
Cntnap2−/− mice	Cortical dysplasia-focal epilepsy syndrome	CNTNAP2 (7q35)	Social interactions impairments, stereotypic behavior, sensory sensitivity, elevated motor activity, nest-building impairments. Normal olfactory discrimination and seizures.	Cook and Scherer, 2008; Scott-Van Zeeland et al., 2010; LaSalle et al., 2015; Piochon et al., 2015
Tsc1+/− mice Tsc2+/− mice	Tuberous Sclerosis	TSC1 (9q34) TSC2 (16p13)	Social interactions deficits, normal olfaction. No difference in motor and sensory functions. Repetitive/restricted behavior or interest.	Chao et al., 2010; Willem Verhoeven, 2011; Ergaz et al., 2016
Viaat-Mecp2 conditional mutant mice	Rett syndrome	MeCP2 (Xq28)	Repetitive and stereotypic/restricted behaviors, abnormal gait and reduced anxiety, irregular breathing, reduced pain and normal olfactory discrimination.	Amir et al., 1999; Chao et al., 2010; Katz et al., 2012; Kron et al., 2012; Samaco et al., 2013
Shank3B mutant mice Shank3A mutant mice Shank3(e4-9) mutant	Phelan-McDermid syndrome	SHANK3 (22q13)	Restricted interest but no repetitive behavior, social interactions, learning, sensory, cognitive and motor deficits.	Giza et al., 2010; Phelan and McDermid, 2012; Ergaz et al., 2016
Ube3A−/− mice (transgenic mice with triple dosage of Ube3A)	Angelman syndrome	UBE3A (15q11)	Impaired social interactions, repetitive behavior and restricted interest.	Kishino et al., 1997; Matsuura et al., 1997; Buiting et al., 2016
TS2 mice	Timothy syndrome	CACNA1C (16p13)	Social interactions impairments, repetitive/stereotypic behaviors, and increased fear conditioning.	Chao et al., 2010; Willem Verhoeven, 2011; Ergaz et al., 2016
PTEN mutant mice	Not defined	PTEN (10)	Social interactions deficits, restricted interest, sensory sensitivity, elevated anxiety, normal motor activity and seizures.	Cook and Scherer, 2008; LaSalle et al., 2015; Piochon et al., 2015

**Table 4:** Preclinical representation of several H3R antagonists in different animal models of SCH.

**Ligand**	**Dose (mg/kg)**	**Species**	**Pharmacological effect observed**	**References**
Thioperamide	10 and 30 (i.p.)	Mice	Increase of prepulse inhibition	Browman et al., 2004; Sadek et al., 2016
Ciproxifan	1, 3, and 10 (i.p.)	Mice	Increase of prepulse inhibition	Browman et al., 2004; Sadek et al., 2016
ABT-239	0.3-3.0 (i.p.)	Rats	Decrease of cognitive deficits induced by ketamine and MK-801	Brown et al., 2013; Sadek et al., 2016
A-431404	0.3-3.0 (i.p.)	Mice	Decrease of cognitive deficits induced by ketamine and MK-801	Browman et al., 2004; Sadek et al., 2016
SAR110894	0.3-3 (p.o.)	Mice	Decrease in Impaired social behaviors	Griebel et al., 2012; Sadek et al., 2016
	0.3-1 (p.o.)	Rats	Decrease in Impaired social behaviors	Griebel et al., 2012; Sadek et al., 2016

### Non-genetic animal models of ASD

Environmental animal models are induced by prenatal exposure of pregnant animals to certain chemical compounds, infections, or inflammations. The environmental inducing factor is only of significance if the chemicals used in the animal model cause the same effects in humans. Accordingly, the antiepileptic drug, VPA was found to significantly increase the ASD degree in offspring of treated mothers (Christianson et al., 1994; Moore et al., 2000), and similarly showed to induce ASD-like behaviors in mice and rats (Rodier et al., 1997; Anshu et al., 2017; Nicolini and Fahnestock, 2017). Therefore, the ASD-like animal model induced by in utero exposure to the anticonvulsant drug VPA has been well established and recognized to study ASD features (Nicolini and Fahnestock, 2017). Moreover, the immunomodulatory drug thalidomide, which was used by pregnant women in the 1950s, was revealed to be associated with a marked escalation in the incidence of ASD in their offspring (Strömland et al., 1994). Furthermore, several reports revealed that methylmercury, which is formed from inorganic mercury by the action of microbes that live in aquatic systems including lakes, rivers, wetlands, sediments, and soils, exposure during childhood caused a variety of neuropsychological abnormalities, e.g., memory, attention, and language, (Grandjean et al., 1999; Falluel-Morel et al., 2007; Davidson et al., 2011). Also, a previous study has shown that acute systemic administration methylmercury during developmental phases elicited hippocampal cell death, reductions in neurogenesis, and severe learning impairments (Grandjean et al., 1999). Thimerosal was a widely used preservative in numerous biological and drug products since 1930s, including many vaccines. It is a mercury-containing organic compound which contains ethylmercury, a compound supposed to be the cause for several adverse neurodevelopmental deficits, including ASD (Hviid et al., 2003). Also, a previous study in which 10,000 cases have been examined showed that there is a significant association between maternal viral infection in the first trimester and ASD in the offspring (Atladóttir et al., 2010). Indeed, when animals were exposed to the maternal immune activation with polyinosine:cytosine (poly I:C) at embryonic day 9.5, the offspring displayed histological and behavioral abnormalities that resembled ASD (Shi et al., 2009; Hsiao et al., 2012; Garay et al., 2013). On behavioral level, these animals demonstrated impaired sociability, communication differences, and increased repetitive stereotyped behaviors and had smaller brain sizes at birth, followed by macroencephaly in adulthood. In line with these observations, it has been shown that viral infections in the mother can prompt substantial changes in the immune system in both mother and fetus, leading to long-term epigenetic alterations in the offspring (Kong et al., 2012). Consequently, the timing of immune insults during developmental phase may be one source of the heterogeneity in the phenotypic features observed in ASD (Kong et al., 2012). Accordingly, male rats treated with thalidomide/VPA demonstrated immunological changes such as decreased splenocyte proliferative response to mitogenic stimulation, lower thymus weight, and decreased interferon (IFN)-β/IL-10 ratio in peritoneal macrophages, whereas female rats in this study failed to show many of these behavioral and immunological changes, indicating that sex-specific responses to some environmental factors might play a significant role on the phenotypic outcomes of several ASD features (Schneider et al., 2008). Also and subsequent to viral infection, the immune response was found to produce various cytokines, such as interleukins (IL)-1,-2 and -6 which modulate the release of several monoamines such as 5-HT in the hippocampus and other brain regions (Libbey et al., 2005). Indeed, maternal infection was found to lead to elevated levels of cytokines and chemokines including interleukin-1β (IL-β), IL-6, IL-8, and IL-12p40 in the plasma of ASD children, and such increases were reported to be associated with more reduced communication skills and abnormal behaviors (Patterson, 2012). Also, the offspring of infected animal mothers given poly (I:C) exhibited both ASD-like behavioral and neuropathological deficits as described above (Shi et al., 2009; Hsiao et al., 2012; Patterson, 2012; Gadad et al., 2013; Garay et al., 2013). Based on the aforementioned results, environmental influences both *during* and *after* pregnancy can significantly influence the immune system and the developing nervous system to play a role in constructing several neurodevelopmental disorders including ASD.

It should be stressed that experimental animal models (genetically manipulated animal models, animal models obtained by destruction of certain CNS areas, and animal models obtained by using maternal factors) are indispensable for exploring the pathophysiologic causes of brain disorders, e.g., ASD, although they do not reflect the entire state of ASD disease. Moreover, animal models (mostly rodents) are widely used to study the development of cortical neurocircuit, genetic analysis and molecular mechanisms underlying ASD, and the palliative effects of newly developed drugs on core as well as associated symptoms of ASD. Notably, rat has become the most extensively used animals in the context of SCH and ASD neuroscience (Brudzynski, 2013). Accordingly, and as compared to mice, rats provide more experimental advantages including the fact that rats demonstrate richer social behavioral skills, show various types of social behaviors, and use a rich CNS communication system (Brudzynski, 2013). Furthermore, genetic as well as non-genetic rodent models have the capability of being the major objects of pharmaceutical industry in testing drug efficacy, dosage and acute as well as (sub)chronic toxicology.

## Conclusion

The disciplines of neurochemistry, neuroanatomy, neuropsychopharmacology, genetics and molecular biology are encouraged to work simultaneously to further help uncover more details of the biological bases of ASD. It is motivating that numerous genes identified so far in human ASD genetic studies generate several valuable transgenic ASD rodent models, which display both ASD-like abnormalities in neuropathology as well as in behavioral phenotypic features. Moreover, several of the abnormalities involve the dysfunction of various neurotransmitter systems. Furthermore, a variety of multiple genetic and environmental factors induce alterations in brain development, leading to ASDs. Notably, there are associated behavioral abnormalities, e.g., anxiety-like behavior, seizure susceptibility, sensory processing, and motor abnormalities, which have been recognised in both the genetic and non-genetic rodent models of ASDs. Consequently, broader understanding of how the trajectory of patients affected with ASDs fits into several subcategories, with specific endophenotype concepts, will expedite both understanding of the causes of the disorders and can provide insight into specific possible future treatments. Preclinical experimental rodent models are supportive to determine the mechanistic pathways associated with the genetic and environmental targeted insults to the CNS and the pathogenesis of ASD. In recent times these animal models are also continuing to proliferate to assess the potential of different drugs and other related treatment modalities. Consequently, this is of valuable significance, as it may close a gap in our understanding of the effects of therapy and may suggest new and effective methods for ASD treatment. The experimental results observed so far clearly show that ASD involves cascade of complex gene-environmental interactions which requires experts of these research areas to work in collaboration together that may lead hopefully to improved pharmacological interventions, if not to cure but at least to reduce the impact of symptoms of this disorder. The majority of pharmacological interventions to date have focused on antipsychotics, antidepressants and psychostimulants that are the most commonly utilized medication classes for ASD. However, several other drug classes, e.g. SSRIs, glutamate antagonists, and antiepileptics, are currently progressing in clinical trials for their future potential use in ASD. In addition, future research in the ASD area needs to address individual differences within the ASD population in order to better account for etiological and phenotypic heterogeneity, and to emphasize on mechanistic processes as well as neurotransmitter dysfunctions and neurodevelopmental routes rather than outcomes or endpoints of the disorder. Moreover, understanding the high level of psychiatric comorbidities and overlapping features shared with other neurodevelopmental disorders together with integration of different research methodologies (e.g., neuropharmacological, behavioral, and brain imaging measures) could encourage further advances of the current level of knowledge in the field of therapeutic ASD intervention. Furthermore, it is becoming progressively obligatory to prospectively evaluate the co-occurrence of, and commonalities between SCH and ASD at the trait level applying the multifactorial data obtainable from large clinical samples, since undiagnosed co-occurring brain disorders in respective individuals may be the consequence of not receiving suitable facilities, assistances, or even pharmacological treatments. Given the similarities between several brain disorders, e.g., SCH and ASD, misdiagnosis is, also, possible. Therefore, it is imperative that future research efforts account for the heterogeneity of both disorders, and inspect evidence on several levels to categorize endophenotypic markers considering the dimensional nature of such brain disorders. In addition, future studies assessing new pharmacological treatment approaches combined with non-pharmacologic therapies are necessary to ensure that target core behaviors of ASD are properly managed.

## Author contributions

NE and BS: Idea, design, writing, and submission. MA-H, AS, SO, and AS: Substantial contribution to the conception, formulation, and critical revision of the manuscript. All authors gave approval for the final submission of the review and agreed be accountable for all aspects of the work.

### Conflict of interest statement

The authors declare that the research was conducted in the absence of any commercial or financial relationships that could be construed as a potential conflict of interest.
